# Supplementary material for: Ethnic disparities in publicly-available pulse oximetry databases
Source: Commun Med (Lond). 2022 May 27;2:59. doi: 10.1038/s43856-022-00121-8 (PMC9142514; doi:10.1038/s43856-022-00121-8)
Supplement: Supplementary file 2 — Description of Additional Supplementary Files [file 43856_2022_121_MOESM2_ESM.pdf]

Description of Additional Supplementary Files

File Name: Supplementary Data 1

Description: Supplementary Data 1 contains source data for the main figures in this manuscript
